# Effects of functional training on tennis-specific physical fitness and functional movement screen in junior tennis players

**DOI:** 10.1371/journal.pone.0310620

**Published:** 2024-09-19

**Authors:** Wensheng Xiao, Xiaorong Bai, Kim Geok Soh, Yang Zhang

**Affiliations:** 1 School of Physical Education, Huzhou University, Huzhou, China; 2 Department of Sports Studies, Faculty of Educational Studies, Universiti Putra Malaysia, Serdang, Selangor, Malaysia; 3 Independent Person, Windermere, Florida, United States of America; Tokat Gaziosmanpasa University Tasliciftlik Campus: Tokat Gaziosmanpasa Universitesi, TÜRKIYE

## Abstract

**Objectives:**

Functional training mimics the coordinated motions of multiple muscle groups and joints performed during exercise. The purpose of this study was to compare the effects of a 12-week functional training and traditional resistance training on the performance in junior tennis players.

**Methods:**

Trained tennis players (mean age: 16.6 years) were assigned to a traditional training group (n = 20) or functional training group (n = 20). The traditional training group received a resistance training program by their coach, while the functional training group was given Santana’s Racket Sport Program. At baseline, after six weeks, and after 12 weeks (T12), the participants’ tennis-specific physical fitness and functional movement screen (FMS) were evaluated.

**Results:**

At T12, both training improved the values for multistage fitness test, hexagon test, planned agility test, sit and reach, and 20 metre sprint (*p* < 0.05); except the flexibility, functional training provided no additional advantages. At T12, functional training enhanced (*p* < 0.01) all seven components of the FMS, and there is a 100% probability that the total score of the FMS would be enhanced. In contrast, for the traditional training group, shoulder mobility of the FMS decreased (*p* = 0.03), and there was no changes in other FMS components at T12.

**Conclusions:**

Functional training is not only effective in improving tennis-specific physical fitness, but it also provides greater functional movement advantages for junior tennis players compared to traditional resistance training.

## 1. Introduction

Professional tennis becomes more physically demanding [1] and a successful on-court performance relies on a complex interplay of fitness components [2]. Agility and speed can aid tennis players in achieving rapid completion and transformation of various sports forms, as well as precise stroke completion. Flexibility not only reflects the adaptations in joint range of motion to meet the musculoskeletal demands of tennis-specific activities but also addresses the potential deficits that may lead to frequent overuse injuries [3, 4]. As the average elite tennis match lasts approximately 90 minutes [1] and is often played in challenging environments, endurance is another essential quality for tennis success [5]. In other words, without an optimal physical fitness, other components of modern tennis game, such as skills and tactics, will not work as premature fatigue can impair nearly every tennis performance. Therefore, to become a professional tennis player, it is crucial for junior players to maximize their physical fitness development in addition to their skills and game experience.

There is a growing interest in optimal training methods, especially those that prioritize the principle of specificity in a balanced manner to achieve performance development. Fernandez-Fernandez et al. evaluated 5-week neuromuscular training in adolescent players and found that neuromuscular training could improve agility, speed, and power [6]. Kilit and Arslan compared 6-week high-intensity interval training to on-court tennis training and concluded that each training method has unique advantages for adolescent players [7]. A recent study finds that 10 weeks of plyospecific training could improve the agility of U16 players [8]. In general, many training methods have been shown to improve tennis-specific physical fitness. More research is needed to determine the optimal training methods for improving the efficacy of training, both in adolescents and adults [9].

Movement-based exercises, as opposed to focusing exclusively on specific muscular and joint adaptations in isolation, may be more effective to improve fitness. In this regard, functional training stands out as an efficient approach. In the context of high-performance sports, functional training refers to compound movements that mimic muscles and joints functioning in sport-specific movement patterns [10]. Existing research suggests that functional training could enhance various aspects of fitness [11]. In one of the few studies conducted on tennis players, Yildiz et al. found that 8 weeks of functional training improved children’s physical fitness [12]. Such adaptations may also occur in junior tennis players, though its efficacy among trained athletes requires further confirmation.

Meanwhile, modern sports training should place equal emphasis on training effectiveness and injury prevention, whereas in practice, efficacy is often prioritized over risk [13]. A previous study found that functional training may be an effective method for enhancing functional movement [14]. In essence, functional training is performed by simulating the target movement, thereby improving the target movement. Traditional resistance training, on the other hand, is not always multi-articular and multi-planar and therefore may overlook the importance of an athlete’s functional limitations and their ability to perform coordinated functional movements accurately. Tennis necessitates swift and coordinated rotations of multiple muscle groups and joints [15], and its explosive movement patterns necessitate not only superior fitness but also make players susceptible to injuries in extremities and joints. In this regard, it is of interest to evaluate the effect of functional training on functional movement, which has dual implications for performance and injury prevention.

Therefore, this study compared functional training and traditional resistance training on specific physical fitness and functional movement in trained junior tennis players. Here, our emphasis is on the movement performance of the lower extremities, as both sudden and gradual onset tennis injuries predominantly occur in this region of the body among professional players [16]. First, it was hypothesized that functional training could enhance physical fitness more than traditional training. Second, this study aimed to investigate whether trained tennis players can experience comparable benefits in functional movement by engaging in functional training. Third, it was investigated the optimal duration of functional training. Overall, this study aimed to broaden the theoretical and practical foundations for designing an effective tennis strength and conditioning program for developmental athletes.

## 2. Materials and methods

### 2.1. Experimental design

This study was approved by the Ethics Committee of Universiti Putra Malaysia (protocol number: JKEUPM-2020-283). We used a cluster randomized, single-blind, two-independent group study design. The sample size was estimated *priori*. Specifically, we looked at Yildiz et al.’s evaluation of functional training in a group of young children (mean age: 9.6 years) [12]. We extracted their results regarding flexibility, 10-m acceleration, agility, and the functional movement screen (FMS) between the functional training and traditional training groups. Based on those results, we calculated an aggregated effect size. Of note, their data were non-parametric, which makes our estimation of their effect size less robust. Based on Cohen’s *d* = 1.215, alpha = 0.05, and power = 0.95, G*Power 3.1 estimated that 38 players are needed for a two-independent group study.

Participants were recruited from two tennis training bases in Zhejiang province, China. In China, adolescents undergoing structured tennis training are typically supervised in specialized sports training schools. Novice players receive fundamental training in regular public schools, while skilled players have the opportunity to voluntarily train in government-operated sports institutions. Exceptionally talented individuals are selected by provincial teams and may eventually be chosen for the national team. The individuals in our sample population belong to the second tier, receiving training in these professional sports institutions. Using a computer-based lottery method [17], volunteers from two training bases were either assigned to the traditional training group or functional training group. Of note, this group information was blinded to the data analysis researcher (X.B.) until the end of the statistical analysis. Volunteers were informed of the potential risks, benefits, and their rights before the study. In addition to their volunteering participation, the following eligibility criteria must be met: at the time of enrollment, participants must have been under the age of 18; had no recent (within one year) major tennis-specific injury history, such as knee, elbow, or shoulder injury, rheumatoid disease, or neurological damage; and had not performed functional type exercise in the past 12 months. Following the screen, each participant’s legal guardian gave his or her written informed consent.

Due to a shortage of female players in the training bases, only male players were recruited. We assessed a total of 57 volunteers, of whom 44 fulfilled the eligibility criteria and were randomly assigned to one of the two groups. All athletes participated in national or provincial-level tennis competitions. Two participants from each group withdrew during the follow-up period, but none of them did so due to a sports injury. During the entire study period, no adverse events were reported. Hence, the data analysis included information from a total of 40 participants, with 20 participants in each group. Their baseline demographics were homogeneous between the groups and details (mean ± SD) are as follows: age, traditional training (TT) = 16.5 ± 0.6 yrs, functional training (FT) = 16.7 ± 0.4 yrs; height, TT = 176.4 ± 2.4 cm, FT = 176.2 ± 2.6 cm; body weight, TT = 71.8 ± 3.2 kg, FT = 71.6 ± 3.0 kg; and, tennis training experience, TT = 58.0 ± 4.2 months, FT = 57.9 ± 4.4 months.

Participants began the 12-week intervention in addition to their routine on-court training. The coaches of the traditional training group designed a resistance training program, which is outlined in Table 1. While the micro-cycle training plan was adjusted (e.g., concurrently increase and decrease sets of various exercises) from day to day based on the coach’s experience, it typically utilized resistance training devices to strengthen tennis-specific muscle groups. Meanwhile, the functional training group followed a standardized Santana’s Racket Sport Program [18] as outlined in Table 2, which was administrated by their coach. During the 12-week intervention, coaches in both groups received advice to allocate 60 minutes per session, three times per week (i.e., between 1600–1700 hours on Monday, Wednesday, and Friday) to strength and conditioning training, resulting in comparable cumulative volume.

**Table 1:** Traditional resistance training program.

Duration	Stage 1	Stage 2	Stage 3
Week 1–4	Light jogging Arm circles Wrist flexes Shoulder rotations	Chest press Body-weight squat Push up Sit up Leg curl Knee lifts	Upper body stretch Shoulder stretch Waist stretch Lower body stretch
Week 5–8		Shoulder press Roll up Squat jump Push up bicep curl Leg curl Knee lifts	
Week 9–12		Chest and shoulder press Roll up Split squat Push up bicep curl Lunge jump Standing calf raise	

**Table 2:** Santana’s functional training program.

Triplexes	Stage 1	Stage 2	Stage 3
Conditioning	MB wood chop	MB ABC squat	Rope circles
(week 1–4)	Side T plank	BP staggered-stance fly	Vibration blade throw
	BP compound row	BP staggered stance CLA row	
Strength	BP low-to-high chop	DB or KB lateral reaching lunge	X-up
(week 5–8)	DB single-arm diagonal fly rotation	T push up	SB rollout
		DB or KB staggered-stance bent-over single-arm row	Rope circles
			Vibration blade throw
	BP staggered stance CLA compound row		
Power and endurance	DB or KB lateral reaching lunge	BP high-to-low chop	Single-leg CLA anterior reach
		MB overhead side-to-side slam	
	Skater	BP swim	Rope circles
(week 9–12)	BP low-to-high chop	MB overhead slam	Vibration blade throw
	MB rotational throw: perpendicular	BP high-to-low chop	
		MB overhead side-to-side slam	

Note. BP: bands or pulleys; CLA: contralateral arm; DB: dumbbell; KB: kettlebells; MB: medicine balls; SB: stability balls.

### 2.2. Outcome assessment

Training effects were assessed by two types of tests. First, this study implemented the FMS to assess the functional movement of tennis players. Previous research has shown the usefulness of the FMS as a tool for assessing the quality of functional movement in athletes [19, 20]. Briefly, the FMS consists of seven basic movement patterns: deep squat, hurdle step, in-line lunge, shoulder mobility, active straight leg raise, trunk stability push-up, and rotary stability. Each test is graded on a four-point scale, and the lower score is used for tests that assess both the left and right sides. A score of three indicates the capacity to execute the functional movement pattern without making any compensations. If an athlete performs the movement with compensations, a score of two is assigned. An athlete receives a score of one if they are unable to perform the movement according to published guidelines, and a score of zero is designated for those who experience pain with the movement. Seven basic components can be added to produce a total score. Second, the tennis-specific physical fitness was evaluated using the International Tennis Federation test battery [21], including the multistage fitness test, hexagon test, planned agility test, sit and reach test, and 20 metre sprint. The tests provided are widely used to evaluate the physical fitness of tennis players [22].

The tennis-specific physical fitness and FMS were assessed at baseline (T0), six weeks into the intervention (T6), and at the end of the study (T12). After a basic warm-up, the participants underwent an evaluation of their FMS, which was then followed by an assessment of their tennis-specific physical fitness. The following steps were taken to prevent non-experimental factors from contaminating the outcome assessments. First, on the day of recruitment, all volunteers took a familiarization trial. Second, coaches were instructed to refrain from assigning strenuous exercises 48 hours before each assessment. All participants were required to consume standard meals in the dining hall of the training base 24 hours before the test. Participants were instructed to get adequate sleep the night before the assessment day and to stay properly hydrated the night before and the morning of the assessment day. Alcoholic beverages were strictly prohibited under all circumstances. Third, all tests were administered at the same time of day (i.e., between 08:00 and 11:00) and in the same order. Because there was only one FMS level-2 certified coach in the research team (W.X.), the functional training group completed all assessments on Saturday, while the traditional training group completed theirs on Sunday.

### 2.3. Statistics

The de-identified data that support the conclusions of this study are available on figshare (DOI: https://doi.org/10.6084/m9.figshare.23812122.v1). Two statistical procedures were employed to tackle specific research aims. We used a constrained longitudinal data analysis model [23] for the analysis of tennis-specific physical fitness. In comparison to ANCOVA or repeated-measures ANOVA, constrained longitudinal data analysis focuses on the interaction between treatment and time in randomized controlled trials, and simulation studies demonstrate its superiority in clinical study design [24]. This analysis was performed using the R version 4.3.1. The primary focus of the FMS is the cutoff point (see also Discussion), while the baseline effect is less important. To compare the treatment effect across time, we used the Mann-Whitney test and corrected multiple comparisons using the Holm-Šídák method. This analysis was performed using the GraphPad Prism version 9.0.0. When there was no significant interaction between treatment and time, the single-arm time effect was analyzed using either the Welch ANOVA with Dunnett’s T3 multiple comparison test or the Kruskal-Wallis test with Dunn’s multiple comparison test for non-Gaussian data. The null hypothesis was rejected at *p* < 0.05. To facilitate the dissemination of our research findings, we interpreted the effect size using the probability-based common language effect size [25] and its non-parametric variant [26].

## 3. Results

**Fig 1:**
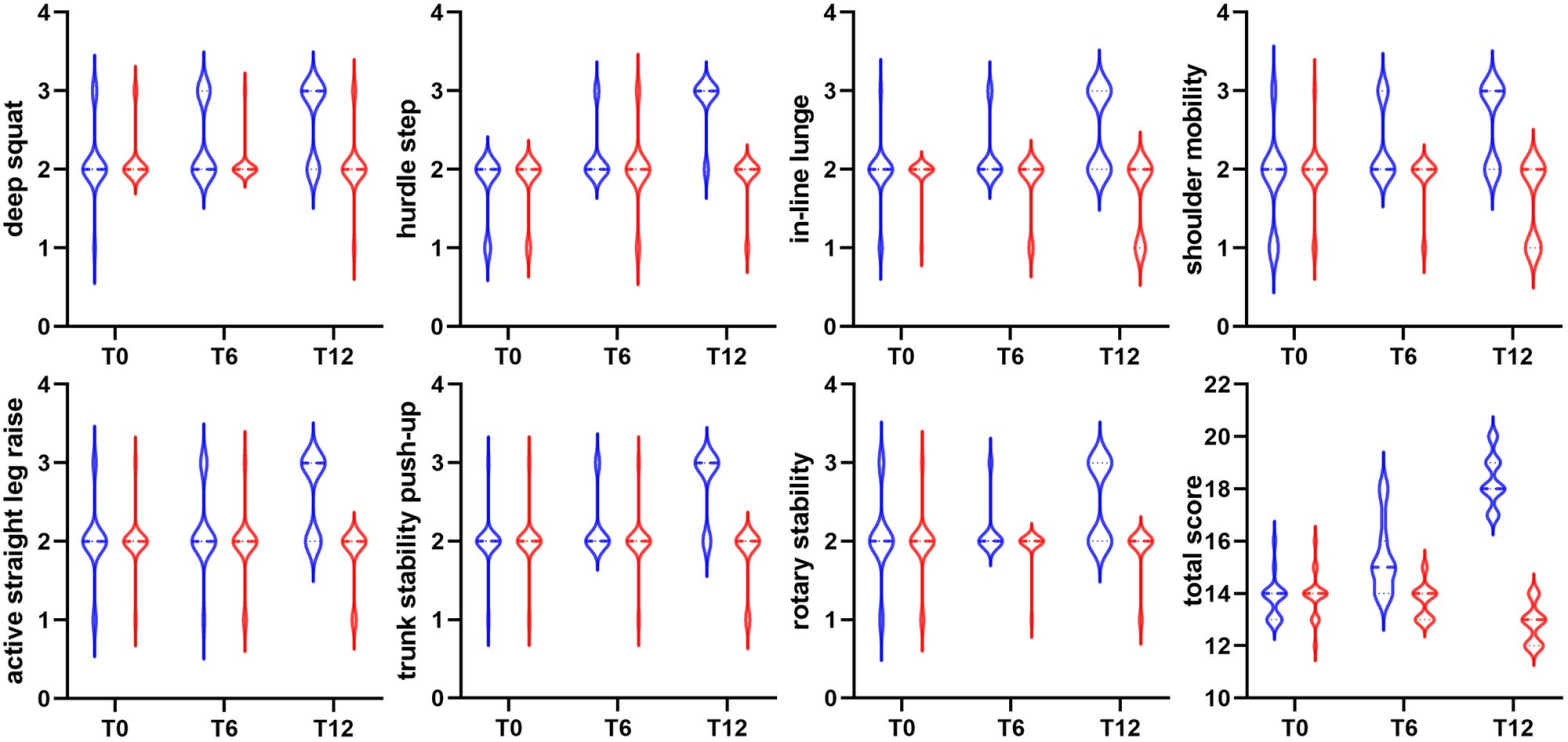
Effects of 12-week training on the functional movement screen. Data in blue color represent the functional training group and data in red color represent the traditional training group.

**Fig 2:**
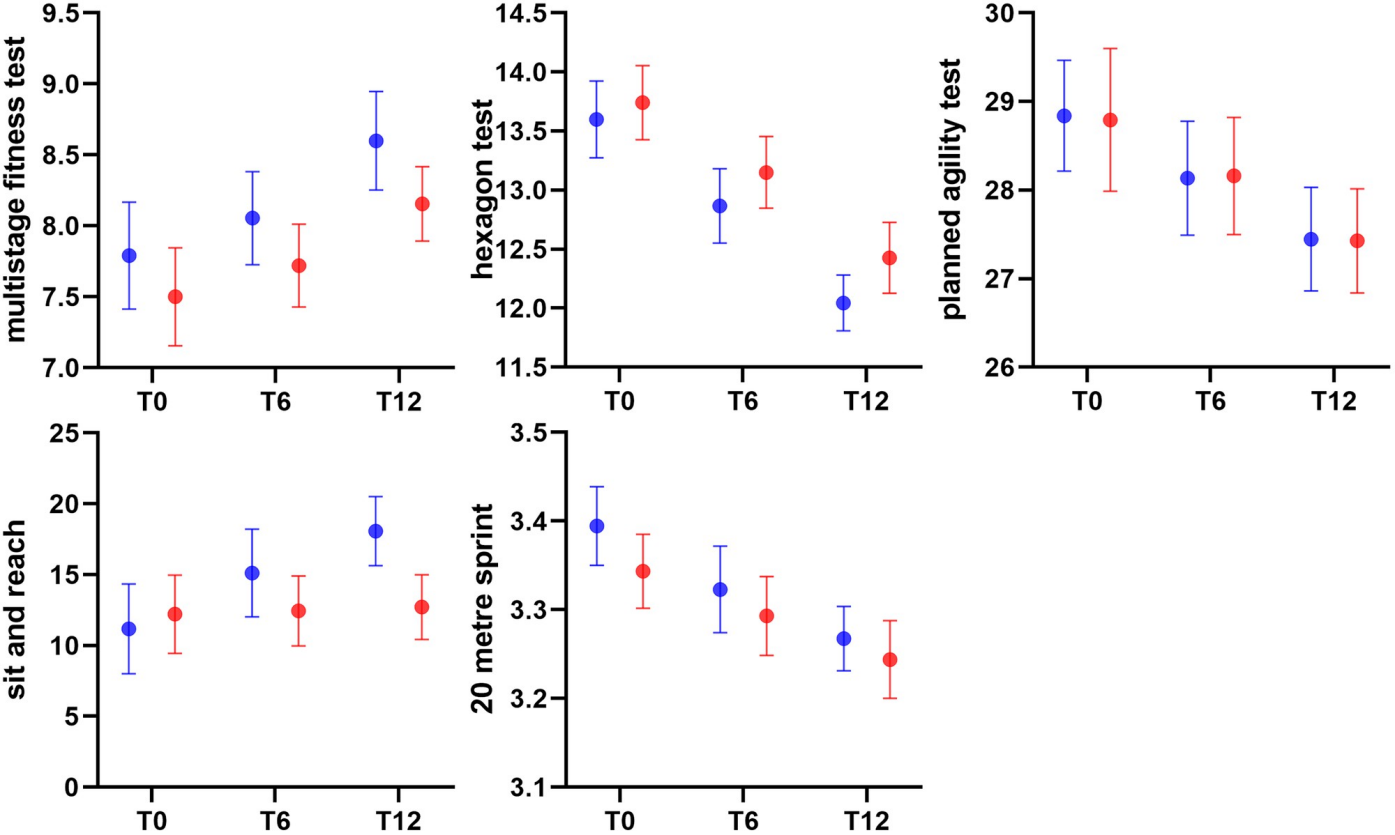
Effects of 12-week training on the tennis-specific physical fitness. Data in blue color represent the functional training group and data in red color represent the traditional training group. The error bars represent a 95% confidence interval.

At T12, functional training outperformed traditional training in every FMS component (Fig 1). According to the effect size presented in Table 3, a random male junior tennis player who engages in functional training has a 100% probability of performing better on the FMS (i.e., total score) after 12 weeks than a random male junior tennis player who engages in traditional training. Except for the sit and reach test at T12, there was no difference between the two groups in terms of tennis-specific physical fitness (Fig 2).

**Table 3:** Summary of significant treatment × time effect.

Measurement	Time	*p*	*A*_*W*_ (%)	CL (%)
deep squat	T12	0.0017	78.4	-
hurdle step	T12	<0.001	93.3	-
in-line lunge	T12	<0.001	82.5	-
shoulder mobility	T6	0.0136	68.5	-
	T12	<0.001	88.0	-
active straight leg raise	T12	<0.001	83.0	-
trunk stability push-up	T12	<0.001	89.4	-
rotary stability	T12	<0.001	77.5	-
total score	T6	<0.001	87.5	-
	T12	<0.001	100	-
sit and reach	T12	0.0053	-	77.4

Note. *A*_*W*_—*A*_*W*_ statistic; CL—common language effect size.

Table 4 presents the single-arm time series effect size. The majority of individual effects did not become statistically significant until the 12-week mark. The traditional training group showed a decline in the shoulder mobility at T12. Physical fitness adaptation occurred as early as six weeks for the hexagon test, and at T12, both training programs improved tennis-specific physical fitness.

**Table 4:** Summary of significant single-arm time effect.

Measurement	Group	Time	*p*	*A*_*W*_ (%)	CL (%)
deep squat	FT	T0 vs. T12	0.003	75.9	-
hurdle step	FT	T0 vs. T12	<0.001	94.0	-
in-line lunge	FT	T0 vs. T12	<0.001	75.0	-
shoulder mobility	FT	T0 vs. T12	<0.001	80.0	-
	TT	T0 vs. T12	0.03	66.5	-
active straight leg raise	FT	T0 vs. T12	0.001	77.0	-
trunk stability push-up	FT	T0 vs. T12	<0.001	85.6	-
rotary stability	FT	T0 vs. T12	0.01	70.0	-
total score	FT	T0 vs. T6	0.01	85.5	-
	FT	T0 vs. T12	<0.001	100	-
multistage fitness test	FT	T0 vs. T12	0.006	-	77.0
	TT	T0 vs. T12	0.009	-	76.1
hexagon test	FT	T0 vs. T6	0.005	-	77.5
	FT	T0 vs. T12	<0.001	-	96.5
	TT	T0 vs. T6	0.02	-	73.6
	TT	T0 vs. T12	<0.001	-	92.1
planned agility test	FT	T0 vs. T12	0.005	-	77.6
	TT	T0 vs. T12	0.02	-	73.8
sit and reach	FT	T0 vs. T12	0.003	-	79.1
20 metre sprint	FT	T0 vs. T12	<0.001	-	85.0
	TT	T0 vs. T12	0.004	-	78.0

Note. *A*_*W*_—*A*_*W*_ statistic; CL—common language effect size; FT—functional training; TT—traditional training.

## 5. Discussion

We hypothesized, based on previous studies [12, 27], that functional training could be more effective than traditional resistance training on tennis-specific physical fitness. However, the results indicate otherwise. Our traditional training, which is founded on a coach’s decade-long experience in youth training, is effective over the medium term in stimulating the developmental performance of male junior tennis players. Therefore, a tennis coach may choose either method for eliciting tennis-specific physical fitness. The discrepancies between our finding and previous studies may be attributable to the design of training methodologies. Zırhlı and Demirci investigated tennis-specific motor skills of 10–12-year-old girls [27]. After 8 weeks of functional training, the participants evidenced superior adaptations in the 10-meter sprint, vertical jump, flexibility, hand-grip strength, agility T-test, and Wingate test, whereas none of these adaptations was observed in the routine training group. In another study, Yildiz et al. studied under-10-year-old male tennis players [12] and found that 8-week functional training improved the sit and reach test, countermovement jump, 10 meters sprint, agility T-test, and balance. While both studies recruited young children with tennis experience, their observed benefits might be attributed to the training methods employed in the traditional training groups. The routine training in Zırhlı and Demirci’s study, such as “cross forehand rally in the height-increased net” was essentially on-court tennis training [27]. The traditional training prescribed by Yildiz et al. [12] consisted of single-joint movements that engage local muscle groups. In comparison, the objective of our traditional training group was to maximize strength and conditioning development during a competition preparation phase, indicating that an authentic traditional training regimen is sufficient to induce physical fitness in junior athletes. The lack of improvement in the sit-and-reach test of the traditional training group may indicate that the designated training regime lacks specificity for flexibility development, and coaches should make micro-adjustments to the training program with both performance and injury prevention in mind.

In our sample, functional training stands out for its value in the functional movement, which is in line with previous research in tennis [12], martial arts [14], and other sports [28, 29]. In comparison, none of the FMS components was improved in the traditional training group. At T12, their total score, an useful metric for predicting injury, had even decreased by one point. While the lack of improvement shows that traditional training lacks the specificity to address movement patterns, this overall decrease suggests that traditional resistance training that emphasizes intensive exercise loads during rapid body development may cause muscle strain. In particular, muscles that are repeatedly trained with single-joint and single-planar motions may eventually cause repetitive use of soft-tissue injuries in junior athletes [30]. A meta-analysis revealed that athletes who scored 14 or lower on the FMS were 2.74 times more likely to sustain an injury during subsequent activity than those who scored above 14 [31]. The result from the traditional training group should alert coaches to train athletes according to the principles of functional training.

It is worth mentioning that, except for the hexagon test, none of the adaptations occurred in 6 weeks, suggesting that longer periods, such as 12 weeks in this study, may be necessary to illicit improvement in trained junior tennis players. Neuromuscular adaptations show distinct patterns between untrained and trained populations [32]. Trained athletes, such as those in this study, are more likely to exhibit latent adaptation at the outset and perform better over time than untrained individuals [33].

The present result also adds another data point to the dose-response relationship for the FMS. In our sample, 6 weeks of functional training significantly increased the total score by one point (note that the emphasis is on the cutoff point rather than absolute change), a result comparable to that of martial artists [14]. By the seventh week, American football athletes’ total score on the FMS had increased by three points [28]. And our 12-week analysis reveals a four-point improvement. In this regard, we are cautiously optimistic about the long-term utility of functional training for addressing mobility deficits in developmental athletes.

Several limitations apply to this study. This age cohort was experiencing a growth spurt and hence, normal physical development had an effect on the outcome assessments. In addition, it is imperative to inform coaches that the validity of FMS in injury prevention is not supported by all research. The presence of negative evidence [34], such as the absence of evidence for scalar invariance and uniqueness invariance [35], serves as a cautionary reminder that further refinement of the test may be necessary.

## 6. Practical implications

Combining functional training and regular FMS can be an especially useful integrated program for training junior tennis players. It is advised to regularly (e.g., on a monthly basis) carry out assessments of physical fitness and FMS. Implementing this approach can assist coaches in adjusting their training programs to match junior players’ abilities at various stages during their physical development. It is worth noting that we adopted a total score of 14 on the FMS as the cutoff point of heightened susceptibility to sports injuries [36, 37]. While there may be ongoing debate surrounding the validity of this cutoff point [38], it is difficult to dispute the overarching point that joint and/or muscular pain always serves as a warning signal of sports injury risks. Alemany and colleagues proposed that the incidence of pain may serve as a more robust predictor of injury risk compared to a FMS total score of less than 14 [39]. Therefore, it is advised to consider all seven independent components when interpreting a complete FMS. If an athlete rates a score of zero in any of the individual components, indicating clear pain in the examined body location, coaches should adopt a cautious training program, regardless of whether the total score falls below 14. Overall, combining functional training with routine FMS has the potential to the early identification of junior athletes who are prone to experiencing overtraining syndromes.

## 7. Conclusions

This study aimed to compare the growing popular functional training for developing tennis-specific physical fitness and functional movement in trained junior tennis players. A well-designed traditional resistance training program is effective in improving tennis-specific physical fitness. Nevertheless, musculoskeletal groups are not always trained in a manner that regards the coordinated movement of the entire kinetic chain during sport-specific activity. We show that 12 weeks of functional training illicited similar benefits in physical fitness as traditional resistence training, while also leading to a significant enhancement in FMS scores. Modern sports training should take into account both high performance and injury prevention. Collectively, our results suggest that functional training is effective in developing tennis-specific physical fitness and improving mobility and stability along the kinetic chain of junior tennis players.
